# Impact of heart rate on reproducibility of heart rate variability analysis in the supine and standing positions in healthy men

**DOI:** 10.6061/clinics/2019/e806

**Published:** 2019-08-06

**Authors:** Carlos Janssen Gomes da Cruz, Luiz Guilherme Grossi Porto, Paloma da Silva Rolim, Deleon de Souza Pires, Giliard Lago Garcia, Guilherme Eckhardt Molina

**Affiliations:** I Grupo de Estudos e Pesquisas em Funcao Autonomica Cardiaca (GEFAC), Centro Universitario Euro Americano - UNIEURO, Brasilia, DF, BR; II Laboratorio de Fisiologia do Exercicio, Faculdade de Educacao Fisica, Universidade de Brasilia, Brasilia, DF, BR

**Keywords:** Autonomic Nervous System, Parasympathetic Nervous System, Reliability, Test-Retest Reliability, Posture, Heart Rate

## Abstract

**OBJECTIVE:**

The reliability of heart rate variability (HRV) analysis is not yet fully understood, especially considering different body positions and the mathematical influence of heart rate. The aim of this study was to evaluate the reliability of HRV in supine and standing positions, with and without mathematical adjustment of HRV by the average R-R interval (iRR).

**METHODS:**

We evaluated 37 young males (23.1±4 years; 25.1±3 kg/m^2^). A 5-min segment of the iRR was collected in the supine and standing positions on three occasions separated by 48-hour intervals. Absolute and relative reliability of temporal and spectral indices were assessed by the coefficient of variation (CV) and the intraclass correlation coefficient (ICC), respectively.

**RESULTS:**

We did not observe differences in HRV indices in the three occasions in the supine or standing position (*p*>0.05). Moderate to good reproducibility was observed for temporal and spectral indices of HRV in the supine position (ICC: 0.65-0.89; CV: 0.9-19.8). In the orthostatic position, low to good reproducibility was observed (ICC: 0.35-0.89; CV: 1.1-34.8), with higher ICCs for temporal indices. After mathematical adjustment, only a small modification in HRV reliability was observed in both positions.

**CONCLUSIONS:**

In young adult males, the mathematical adjustment of HRV by the average iRR led to a nonsignificant effect on HRV reliability. Additionally, HRV reliability is dependent on body position and the index analyzed. Promising measures in both supine and standing positions include r-MSSD and the HF band (parasympathetic indices).

## INTRODUCTION

Heart rate variability (HRV) consists of fluctuations in the intervals between successive heartbeats, defined by the distance between two R waves 1. Analysis of HRV is a valid and noninvasive method widely used to evaluate the effect of pharmacological and nonpharmacological interventions on cardiac autonomic function 2-4

The clinical relevance of HRV assessment has been shown in several populations. Low resting HRV is associated with a negative prognosis in healthy individuals 5 and in several clinical conditions such as diabetes 6, hypertension 7, cancer 8, myocardial infarction 9 and others 10,11. In sports science, HRV is usually used to evaluate the acute 12,13 and chronic 4 effects of exercise training on cardiac autonomic modulation, which may be a useful strategy for monitoring exercise-induced “internal underload” and to prevent nonfunctional overreaching episodes 14.

Due to the relevance of HRV analysis in clinical and sports areas, knowledge about the interday reliability of this measurement is mandatory for adequate interpretation of the possible effects of different interventions in cardiac autonomic modulation 12,15,16. In this scenario, interday HRV reliability has been usually evaluated in a single body position 15,17,18, which may be a limited approach because the autonomic effects of different pharmacologic and nonpharmacologic interventions have been assessed in different body positions and functional conditions 19,20. Thus, studies investigating the absolute and relative reliability of different HRV indices in different body positions can substantially contribute to cardiac autonomic analysis in clinical and scientific settings.

Another aspect that deserves attention is the possible influence of heart rate (HR) on HRV. It has been demonstrated that reductions in HR can mathematically increase HRV, unrelated to autonomic mechanisms 22,23, and modify the prognostic power of HRV indices 21. This issue should also be considered in sports science because exercise training can reduce HR by nonautonomic mechanisms 24,25 and promote nonphysiological changes in HRV. Therefore, adjusting HRV indices for the corresponding average iRR (iRR_average_) might minimize (or exclude) inherent mathematical bias and allow a more precise clinical and functional interpretation of HRV values 21,26, as elegantly demonstrated by pharmacological blockade and bilateral cervical vagotomy 27.

Because the adjustment of HRV by iRR_average_ appears to be an important approach for improving HRV analysis and because the amplitude and variation of iRR are dependent on body position, it is essential to evaluate the impact of these two variables in the HRV reproducibility analysis because poor reproducibility may lead to erroneous inference about the subject's physiological status. Therefore, we aimed to evaluate the reproducibility of temporal and spectral analysis of HRV, with and without mathematical adjustment by iRR_average_, in healthy male subjects in resting supine and standing positions. Based on previous studies with different methodological approaches and populations 28,29, we hypothesized that HRV reliability is more dependent on the body position and the analyzed index than on the iRR_average_ itself.

## MATERIALS AND METHODS

### Participants and ethical issues

We evaluated 37 young male subjects. The inclusion criteria were participants 20 to 30 years old, nonsmokers, without any cardiovascular or other disease or symptomatic clinical manifestations, without muscle or joint disorders, and not taking any medications. Those who did not follow the recommendations or started drug treatment during the data collection period were excluded (n=3).

Before the beginning of the tests, all participants provided written informed consent with approval of the Ethical Committee on Human Research of UNIEURO, in compliance with the Brazilian National Research Ethics System Guidelines and Declarations of Helsinki.

### Baseline data

First, we recorded anthropometrical (body mass and height) data, basic lifestyle habits and clinical data, smoking and alcohol consumption habits, symptoms and/or diagnoses of chronic disease and physical activity level. The physical activity level was assessed using the International Physical Activity Questionnaire-IPAQ 30, and all other information was obtained by clinical anamnesis. After 10 min of resting in the supine position, blood pressure, heart rate (HR_baseline_) and respiratory rate were recorded. All measures were performed in a quiet, clinical laboratory room at controlled ambient temperature (21°C-24°C) between 2:00 and 5:00 p.m. Participants were oriented to abstain from stimulants, alcoholic beverages, medicine and physical activity for at least 48h prior to evaluations. The study design consisted of three visits to our laboratory at intervals between 48 and 72 hours; at each visit the participants were subjected to a standardized HRV analysis.

### Resting heart rate variability

Following 10 min of rest in the supine position, a 5-min series of iRR was recorded according a standardized protocol 20,31. Subsequently, subjects were asked to actively adopt the orthostatic posture at bedside, and after 3 min in this position, an additional 5-min series of iRR was recorded. The subjects continued to breath spontaneously and regularly; their respiratory rate was visually monitored, and breaths were counted.

The iRR series were obtained by a model V800 Polar cardiac monitor^®^ 32 and analyzed using Kubios HRV analyzer software (MATLAB TM version 2.0, Kuopio, Finland). After a beat-to-beat visual inspection, spurious beats were deleted (<1% of total iRR segment) from the series without adding new intervals 31.

The time-domain indices measured were the root mean square of successive differences between the adjacent normal iRR (r-MSSD) and the standard deviation of normal iRR (SDNN). In the frequency domains, we calculated the absolute power of low (LF-0.04-0.15 Hz) and high (HF-0.15-0.50) frequency bands and the normalized power areas of low (LF_n.u_) and high frequency (HF_n.u_) bands, which were expressed as percentages and calculated as the absolute power area of each band divided by the sum of both absolute areas multiplied by 100.

From a physiological perspective, the r-MSSD and the HF band primarily exhibited parasympathetic modulation, and the SDNN and the total power spectral area exhibited global modulation (sympathetic and parasympathetic influence); the LF band was previously called the “baroceptor band” because it primarily reflects baroceptor activity while at a rest 33.

### Mathematical adjustment for HRV analysis

To minimize any mathematical bias from the HRV analysis, absolute temporal and spectral indices were corrected by division with the corresponding mean iRR (ms) or mean iRR (s)^2^, respectively 23,27. Because normalized spectral indices reflect the relative contribution of the HF and LF bands in the spectral area and not the power of these indices, mathematical adjustment by iRR_average_ is inadequate and was not adopted for these markers in the present study.

### Statistical analysis

Based on the results of the Shapiro-Wilk test, the hypothesis of normality was violated in many variables. Thus, logarithm transformation was realized for the reliability analysis 34. Descriptive statistics were performed based on the median and interquartile range, and comparisons among the three trials were made using a nonparametric Friedman test. Differences among the three trials were identified by a Duncan post hoc test. The data were also analyzed without logarithm transformation for better visualization of the magnitude of the variables.

The sample size was defined based on the study objectives. As shown by Pinna et al. (2007), the sample size required for reliability studies involving resting HRV analysis is dependent on the indices evaluated, ranging from 24 to 57 individuals for temporal and spectral analysis. Because we adopted eight indices and larger or smaller sample sizes can enhance type I or type II error, respectively, we adopted a sample size between these two extremes (n=37).

Relative reliability was assessed with an intraclass correlation coefficient (ICC) (two-way mixed) and was considered excellent when the ICC was higher than 0.90, good when it ranged from 0.75 to 0.90, moderate when it ranged from 0.50 to 0.75 and poor when it was less than 0.5 35. Absolute reliability was assessed with the intrasubject coefficient of variation (CV). CV is a measure of discrepancy and is expressed as a percentage of the mean (CV=standard deviation / mean X 100). This measure expresses the relative magnitude of individual variation on different indices of HRV considering the three trials. Data were analyzed using SPSS v20 (SPSS Inc., USA).

## RESULTS

The participants in this present study, aged 23.1±4 years, had a body mass index (BMI) of 25.1±3 kg/m^2^ and presented moderate (69.8%) to high (30.2%) physical activity levels according to the IPAQ.

There was no difference in resting HR, systolic and diastolic blood pressure or respiratory rate among the three trials (*p*>0.05) (Table 1). Similarly, no difference was observed in any of the HRV indices in the supine or standing position (Table 2).

In the supine position, the ICC range was from 0.65 to 0.89, and the CV range was from 0.9 to 19.8 (Table 3). After mathematical adjustment, the ICC range varied from 0.60 to 0.81, and the CV ranged from 6.1 to 11.9 (Table 4).

As shown in Table 3, the ICC during orthostatic stress ranged from 0.35 to 0.89, and the CV varied from 1.1 to 34.8. After mathematical adjustment, the ICC ranged from 0.32 to 0.88, and the CV ranged from 7.1 to 16.9 in this position (Table 4).

## DISCUSSION

Confirming our initial hypothesis, the results of the present study show that mathematical adjustment of HRV by iRR_average_ does not induce a significant effect on the reliability of time-domain and power spectral indices of HRV. Additionally, low to high reliability of HRV analysis was observed according to the index investigated and body position adopted to record the iRR segment.

In the supine position, we observed moderate to high relative reproducibility in the time-domain (ICC=0.65-0.89) and power spectral (ICC=0.65-0.83) indices of HRV, without expressive differences between methods. After mathematical adjustment, a small modification of reliability was observed. In the orthostatic position, a high ICC was observed for time-domain indices (ICC=0.86-0.89), and the adjustment by iRR_average_ promotes a slight variation of these values (0.87-0.88). In the power spectral analysis, the HF and LF bands showed high (ICC=0.83) and low (ICC=0.35) relative reliability, respectively. After adjustment, only a small modification of relative reliability was observed in these markers.

Our findings show greater relative reliability of time-domain indices of HRV in the orthostatic position. On the other hand, a lower ICC was observed for the LF band in this body position, with no changes in the HF spectral area. In fact, previous studies of young men revealed a higher reproducibility of time-domain analysis than spectral analysis 36 and low variation in HRV indices in the orthostatic position 29. However, the greater relative reliability of HRV in the standing position was not confirmed in children 28 or in older healthy women 37.

Good absolute reliability was observed for the time-domain and absolute power spectral indices, represented by the low intrasubject CV of these measures in both the supine and standing positions. However, spectral analysis of HRV in normalized units showed larger individual variation (12.3-34.8%). This result should be interpreted with caution because in that case, the CV reflects the relative variation of a relative index (%). For example, an interday variation in the spectral index of normalized units from 30 to 36% results in a variation of 20%, which represents a large mathematical, but not biological, oscillation. The same principle could be applied when the temporal indices were mathematically corrected.

Investigating the influence of mathematical adjustment of HRV by iRR_average_ on the reproducibility of iRR variability is an important approach. Sacha (2014) demonstrated that the division of HRV by the iRR_average_ enhances the prognostic performance of HRV for cardiac and noncardiac deaths in women and for noncardiac death in men. Corroborating these results, Pradhapan et al. (2014) observed in a sample of 1288 patients in the Finnish Cardiovascular Study that the predictive capacity of HRV during rest and post exercise conditions was increased after the mathematical adjustment of HRV by iRR_average_. Thus, these and other studies indicate that the predictive power of HRV parameters for both cardiac and noncardiac mortality can be increased when the HR influence is diminished.

In exercise science, it has been demonstrated that exercise training increases the HRV and, in parallel, reduces the resting heart rate 4. Thus, exercise-induced changes in HRV occur for mathematical (HR reduction) and physiological reasons, such as due to a shift in the sympathovagal balance through the absolute or relative parasympathetic domain and/or a reduction in the intrinsic discharge rate of the sinus node 20,25. In this scenario, it was elegantly demonstrated by bilateral cervical vagotomy and pharmacological blockade that mathematical adjustments of HRV by iRR_average_ minimize these problems 27 and, according to our results, do not induce relevant effects on the absolute or relative reliability of HRV in the supine or standing positions.

Although some evidence has justified the mathematical adjustment of HRV by iRR_average_, this procedure is an emerging methodological approach and, consequently, deserves future investigation to determine its appropriate applications in clinical and exercise settings. Therefore, we highlight that our objective was to compare the absolute and relative reliability of HRV adjusted by iRR_average_ to traditional HRV analyses, rather than encouraging indiscriminate adoption of this mathematical adjustment procedure.

Although several studies have investigated the reproducibility of HRV analysis, direct comparison of previous results with our findings is limited because studies have involved different populations 18,28, physiological or functional conditions 12 or amplitudes of iRR segments 15. Additionally, to the best of our knowledge, this is the first study to evaluate the impact of body posture and iRR_average_ on the reproducibility of HRV analysis in young healthy individuals.

Our results have important practical applications. First, because time-domain indices of HRV presented lower CV and greater consistency in ICC in the supine and standing positions, these markers could be more favorable for assessing cardiac autonomic modulation in both postures. Additionally, it is important to note that good relative reliability was observed for the r-MSSD and HF bands in both positions, indices widely used to assess the acute and chronic effects of pharmachological and nonpharmachological 3,4,27 interventions on cardiac parasympathetic modulation.

The main limitation of this study is a restricted sample composed of young healthy and physically active males, which does not allow the extrapolation of results to other populations. Additionally, our sample was composed of normal weight and overweight men, which could suggest different profiles of HRV in the same group 38. However, all participants were involved in cardiorespiratory and resistance training, which induces muscular hypertrophy and decreases the utility of BMI as an indicator of body fat when BMI is less than 30 39. Additionally, the practice of physical training appears to offset the negative effect of BMI on HRV 38.

In summary, our results demonstrate that the adjustment of HRV by iRR_average_ does not promote a significant impact on HRV reliability and that absolute and relative reliability is dependent on the index analyzed and body posture. Finally, we highlight that the reliability of time-domain indices is less sensitive to postural change. Good reliability was observed for the r-MSSD and the HF band in both supine and standing positions, suggesting that these indices may be attractive alternatives for cardiac parasympathetic modulation assessment in both functional conditions.

## CONCLUSION

We concluded that mathematical adjustment of HRV by iRR_average_ promotes only a small and nonsignificant effect on HRV reliability in young healthy males. Nevertheless, absolute and relative reliability are dependent on body position and the HRV index analyzed, with attractive measures of ICC and CV for the r-MSSD and the HF band (parasympathetic indices) in both supine and standing positions.

## AUTHOR CONTRIBUTIONS

Cruz CJG, Rolim PS, Garcia GL, Porto LGG and Molina GE were responsible for the study design. Cruz CJG, Rolim PS, Pires DS and Garcia GL collected and analyzed the data. Cruz CJG, Rolim PS, Pires DS, Garcia GL, Porto LGG and Molina GE were responsible for the data interpretation and manuscript preparation. All authors approved the final version of the manuscript.

## Figures and Tables

**Table 1:** Median and interquartile range of resting hemodynamic variables assessed on three occasions.

Variables	Trial 01	Trial 02	Trial 03	*p
HR_supine_ (bpm)	61 (55; 73)	62 (55; 68)	65 (57; 72)	0.21
HR_standing_ (bpm)	72 (63; 87)	75 (65; 86)	78 (67; 89)	0.11
RR_supine_ (breaths/min)	15 (12; 17)	15 (14; 17)	15 (11; 18)	0.68
RR_standing_ (breaths/min)	16 (13; 17)	16 (14; 18)	15 (13; 17)	0.09
SBP_supine_ (mmHg)	112 (111; 119)	114 (112; 120)	113 (111; 115)	0.14
SBP_standing_ (mmHg)	113 (112; 114)	116 (111; 120)	114 (111; 119)	0.81
DBP_supine_ (mmHg)	75 (69; 80)	72 (65; 80)	72 (66.; 80)	0.22
DBP_standing_ (mmHg)	70 (62; 75)	70 (66; 72)	71 (75; 61)	0.39

* Nonparametric Friedman Test; HR_baseline_: resting heart rate, RR: respiratory rate, SBP: systolic blood pressure, DBP: diastolic blood pressure.

**Table 2:** Median and interquartile range of heart rate variability analysis assessed on three occasions.

Variables	Trial 01	Trial 02	Trial 03	*p
*Supine position*				
Mean iR-R (ms)	988 (872; 1008)	1001 (807; 1045)	958 (854; 1041)	0.09
SDNN (ms)	75.4 (63.8; 96.1)	74.3 (57.3; 95.5)	81.3 (62.9; 94.1)	0.50
r-MSSD (ms)	61.9 (48.5; 86.1)	57.3 (40.1; 93.4)	62.4 (50.3; 89.2)	0.72
LF (ms^2^)	1223 (861; 2319)	1309 (793; 2392)	1484 (1054; 2172)	0.46
HF (ms^2^)	1458 (931; 2894)	1073 (678; 3063)	1516 (1003; 2844)	0.36
LFn.u (%)	47.3 (37.7; 58.1)	50.5 (39.3; 63.1)	50.5 (37.3; 60.2)	0.53
HFn.u (%)	50.7 (41.8; 62.1)	49.3 (36.7; 60.4)	49.3 (39.7; 62.5)	0.56
*Standing position*				
Mean iR-R (ms)	789 (677; 869)	753 (657; 824)	739 (668; 812)	0.12
SDNN (ms)	61.8 (45.1; 78.8)	54.6 (44.3; 73.2)	52.3 (45.8; 72.7)	0.64
r-MSSD (ms)	33.2 (20.1; 44.9)	28.2 (20.2; 34.4)	26.5 (21.6; 33.2)	0.17
LF (ms^2^)	1519 (728; 2856)	1510 (685; 2435)	1446 (1136; 2671)	0.92
HF (ms^2^)	416 (176; 1006)	322 (178; 477)	336 (188; 537)	0.46
LFn.u (%)	80.5 (67.1; 86.7)	83.7 (73.6; 87.5)	82.1 (72.4; 89.1)	0.25
HFn.u (%)	19.4 (13.2; 32.8)	15.6 (11.8; 22.3)	17.9 (10.7; 27.2)	0.56

* Nonparametric Friedman Test. Mean iR-R: mean R–R interval of the series; SDNN: standard deviation of normal iRR; r-MSSD: root mean square of successive differences between the adjacent normal iRR; LF: absolute power of the low frequency band; HF: absolute power of the high frequency band; LFn.u: normalized low frequency band; HFn.u: normalized high frequency band.

**Table 3:** Absolute and relative reliability of temporal and spectral analysis of heart rate variability in the supine and standing positions.

Variables	ICC (95% confidence)	CV% (95% confidence)
*Supine position*		
Mean iR-R_Ln_ (ms)	0.89 (0.82-0.84)	0.9 (0.7-1.1)
SDNN_Ln_ (ms)	0.65 (0.40-0.89)	5.4 (4.1-6.8)
r-MSSD_Ln_ (ms)	0.78 (0.63-0.88)	6.1 (4.3-7.8)
LF_Ln_ (ms^2^)	0.65 (0.40-0.80)	6.3 (4.7-7.9)
HF_Ln_ (ms^2^)	0.83 (0.70-0.90)	6.6 (4.9-8.4)
LFn.u (%)	0.75 (0.57-0.86)	19.6 (15.5-23.6)
HFn.u (%)	0.75 (0.57-0.86)	19.8 (15.1-24.5)
*Standing position*		
Mean iR-R_Ln_ (ms)	0.87 (0.78-0.93)	1.1 (0.8-1.3)
SDNN_Ln_ (ms)	0.89 (0.82-0.94)	4.5 (3.4-5.6)
r-MSSD_Ln_ (ms)	0.86 (0.77-0.92)	7.8 (5.9-9.8)
LF_Ln_ (ms^2^)	0.35 (-0.12-0.64)	9.9 (4.8-15.1)
HF_Ln_ (ms^2^)	0.83 (0.70-0.90)	9.4 (6.3-12.4)
LFn.u (%)	0.41 (-0.10-0.67)	12.3 (7.7-16.8)
HFn.u (%)	0.63 (0.36-0.79)	34.8 (26.6-42.9)

ICC: intraclass correlation coefficient, CV: coefficient of variation. Mean iR-R: mean R–R interval of the series; SDNN: standard deviation of normal iRR; r-MSSD: root mean square of successive differences between the adjacent normal iRR; LF: absolute power of the low frequency band; HF: absolute power of the high frequency band; LFn.u: normalized low frequency band; HFn.u: normalized high frequency band; Ln: data transformed by the natural logarithm.

**Table 4:** Absolute and relative reliability of temporal and spectral analysis of heart rate variability in the supine and standing positions after mathematical adjustment by average iRR.

Variables	ICC (95% confidence)	CV% (95% confidence)
*Supine Position*		
SDNN_Ln_ (ms)	0.60 (0.31-0.78)	11.6 (-5.5-18.7)
r-MSSD_Ln_ (ms)	0.74 (0.56-0.86)	11.9 (-13.4-37.5)
LF_Ln_ (ms^2^)	0.63 (0.36-0.79)	6.1 (-3.1-15.3)
HF_Ln_ (ms^2^)	0.81 (0.68-0.89)	7.4 (-2.8-17.6)
*Standing position*		
SDNN_Ln_ (ms)	0.88 (0.79-0.93)	7.9 (-2.9-18.8)
r-MSSD_Ln_ (ms)	0.87 (0.79-0.93)	16.9 (-16.3-50.2)
LF_Ln_ (ms^2^)	0.32 (-0.15-0.63)	9.3 (-21.9-39.8)
HF_Ln_ (ms^2^)	0.84 (0.73-0.91)	7.1 (-8.3-22.4)

ICC: intraclass correlation coefficient, CV: coefficient of variation. SDNN: standard deviation of normal iRR; r-MSSD: root mean square of successive differences between the adjacent normal iRR; LF: absolute power of the low frequency band; HF: absolute power of the high frequency band; Ln: data transformed by the natural logarithm.
